# Extracurricular activities in medical education: an integrative literature review

**DOI:** 10.1186/s12909-023-04245-w

**Published:** 2023-04-22

**Authors:** Sejin Kim, Hyeyoon Jeong, Hyena Cho, Jihye Yu

**Affiliations:** 1 Office of Medical Education, Ajou University School of Medicine, Suwon, South Korea; 2 Department of Medical Education, Ajou University School of Medicine, Suwon, South Korea

**Keywords:** Extracurricular activities, Undergraduate medical education, Medical students, Type of extracurricular activities, Outcome of extracurricular activities

## Abstract

**Background:**

The importance of extracurricular activities (EAs) has been emphasized in medical education. These activities could enhance medical students’ emotional and physical health and afford them developmental opportunities. Despite the growing amount of research related to this theme, few studies review and synthesize the existing literature. This study aims to provide an understanding of the educational implications of EAs in medical colleges and constructs an integrated conceptual framework concerning their types and learning outcomes by literature review.

**Methods:**

An integrative literature review was conducted following Torraco’s method, with the aim to generate a new framework for the given topic. The authors utilized Scopus and PubMed as databases, using search terms “extracurricular,” “medical,” and “students.” Initially, titles and abstracts were screened to include relevant studies, and the researchers verified the eligibility of the articles by following the inclusion and exclusion criteria. Of the 263 articles identified, 64 empirical studies were selected for further review.

**Results:**

EAs in undergraduate medical education can be classified into direct extracurricular activities and indirect extracurricular activities, the latter of which is sorted into nine sub-categories. We identified seven main categories regarding the learning outcomes of EAs. In addition to general activities (e.g., pro-social activities, team sports), some distinctive activities such as research have been largely addressed in previous studies. The results of EAs were discussed in relation to academic growth, career development, and psychological experiences.

**Conclusions:**

This review identified the types and learning outcomes of EAs in the context of medical education, thereby suggesting ways to improve the quality of EAs and maximize their educational effects.

## Background

The schedules of medical students are characterized by a heavy academic load, frequent tests, and clinical clerkships. They also invest their time and effort in extracurricular activities (EAs). More than 60% of medical students participate in EAs [1, 2] and spend 9.8 h per week on them [3]. EAs are essential in the context of medical education because these activities serve as a buffer against the stress and burnout developed in an academically taxing environment [2, 4]. Additionally, they provide medical students with developmental opportunities to foster self-esteem, build constructive peer relationships, and enhance student agency [5]. With the global inclination toward student-centered education and the idea that the fundamental goal of assessment should be to foster students’ competence and subsequent learning momentum, a criterion-referenced grading system is highly regarded by many educators [6, 7]. The significance of EAs in medical education is growing because it deals with diverse activities to promote personal development across various domains that cannot be cultivated only by the curriculum [8].

The concept of EA has been defined differently by various scholars. However, recent studies reflect a consensus that EAs, as academic or non-academic activities that are conducted under the auspices of the school and occur outside of regular classroom time, are not part of the curriculum [9–11]. Generally, EAs do not involve grading or account for academic credits, and participation is optional and voluntary for students [10].

Previous studies classify EAs based on the characteristics of the activities (see [12, 13]). Instead of classifying EAs based on prescribed norm, researchers tended to group EAs together if they seem to look alike. Therefore, an agreed categorization of these activities does not exist. The Michigan Study of Adolescent Life Transitions (MSALT), a longitudinal study of sixth-graders in Southeastern Michigan from 1983 to 1997, made progress in defining the types of EAs by grouping the activities [14]. Through this study, Eccles and her colleagues (2003) distinguished types of EAs as (1) pro-social activities─church attendance and/or volunteer and community service type activities, (2) performance activities─school band, drama, and/or dance, (3) team sports─one or more school teams, (4) school involvement─student government, pep club and/or cheerleading and, (5) academic clubs─debate, foreign language, math or chess clubs, science fair, or tutoring in academic subjects [14]. Meanwhile, Bartkus and his colleagues (2012) put EAs on a continuum that ranges from direct to indirect [10] rather than grouping EAs into static categories. They defined a direct extracurricular activity (DEA) as “one that is more closely associated with the student’s major or curriculum” and an indirect extracurricular activity (IEA) as “one that is relatively unrelated to the student’s major or curriculum.” [10, p.699]. They evaluated each EA how closely the content of EA related to academics to specify the type of EA. The classification of direct and indirect EAs by Bartkus et al. (2012) is meaningful because it provides a secure conceptual basis to encompass a wide range of activities.

A growing body of research investigates EAs in medical education. However, these are conducted in fragmented ways, which could restrict a holistic understanding of the given topic [10]. For example, there are ample studies about research activities in medical colleges, though they were not limited to the extracurricular programs (for a review, see [15]). The most prevalently investigated outcome of EA participation in colleges is academic achievement (e.g., [11]). Even though EAs are encouraged in medical education, it is difficult to find a comprehensive study that covers the types of activities offered and their effects. A comprehensive model of EAs in medical education which elaborates on their types and outcomes would contribute to a deeper understanding of EAs and medical education.

### Aim

The aim of this study is to review the research and summarize what kinds of EAs are being conducted and their outcomes in medical colleges. This study seeks to present a comprehensive model that contains both EAs types and EA outcomes in medical education.

## Methods

The integrative literature review is a form of research that generates new frameworks and perspectives about the topic by reviewing, critiquing, and synthesizing representative literature in an integrated manner [16]. Torraco (2005)’s method has been widely used in the field of Human Resource Development (HRD) where new topics are constantly emerging, and a holistic conceptualization and synthesis of the literature is needed. This method is particularly useful when a large body of studies has been conducted, but no comprehensive perspective from which to view a certain topic has been developed. We utilized an integrative literature review to capture the dynamics and development and present a holistic view as previous studies on EAs in medical schools become more abundant and diversified [16]. Moreover, considering that this method addresses complex relationships between constructs, utilizing it would be appropriate to deal with dynamics among different types of EAs and their outcomes in medical colleges.

While undertaking initial research in September 2022, the authors utilized the Scopus and PubMed databases, using the keywords “extracurricular,” “medical,” and “students” to identify the most relevant articles. This initial search yielded 221 matching articles, which were pared down using a staged review [16]. A staged review is the process of narrowing down apposite articles and examining them more critically. In most cases, an initial review of abstracts is conducted, and then an in-depth review takes place [16]. Generally, this approach allows researchers to gain more thorough coverage of relevant articles while minimizing the risk of overlooking pertinent information or including irrelevant sources.

**Fig. 1:**
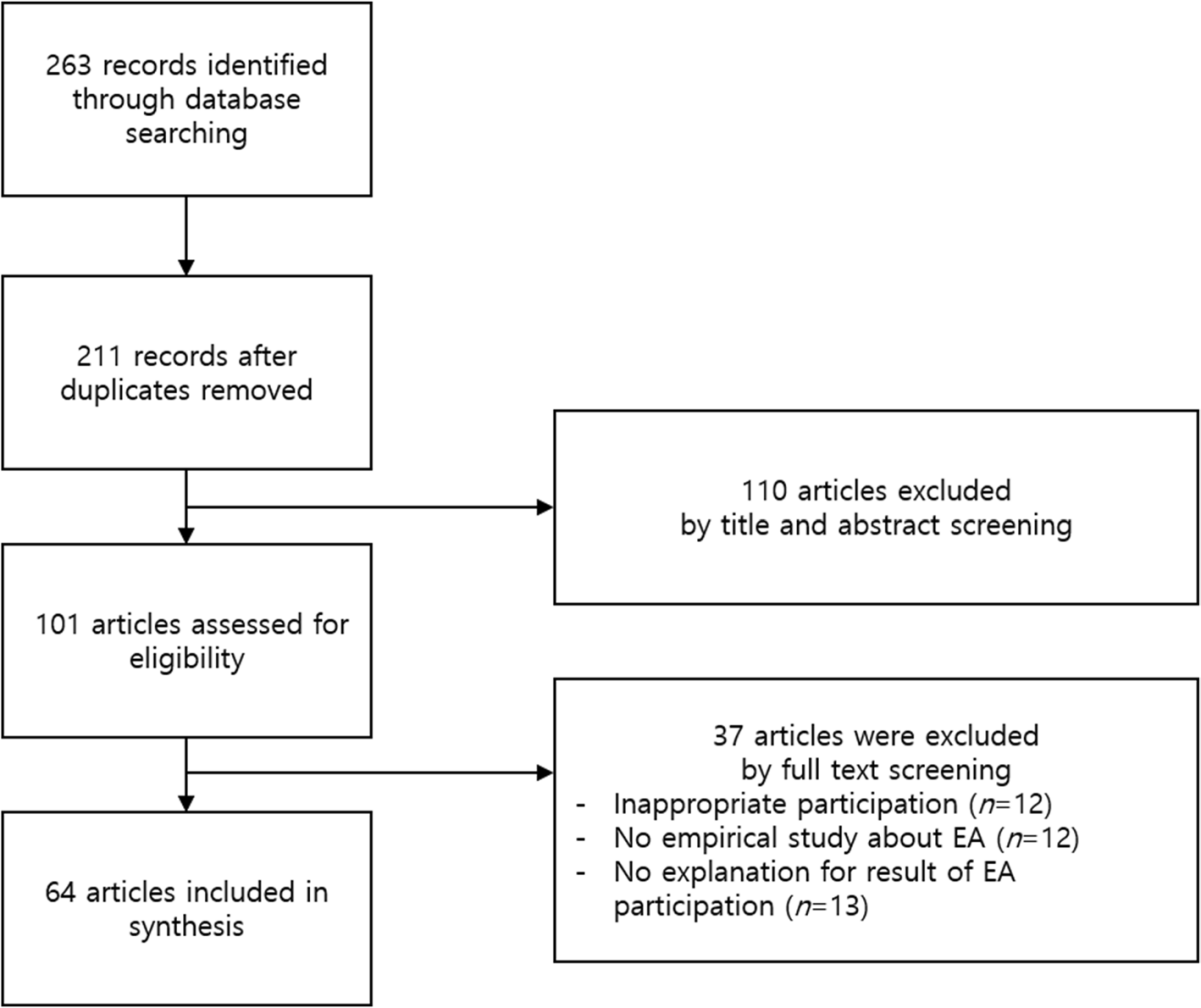
Flow diagram of the screening and selecting processes

The article inclusion criteria required that all articles were peer-reviewed, available in full text, written in English, and published in the last ten years (2013- September 2022). Moreover, we only included those articles elaborating on EAs in undergraduate medical education. The criteria of exclusion are as follows. First, studies with non-undergraduate medical students (e.g., nursing students, veterinary medicine students, etc.) were excluded. Studies about graduate medical students who participated in EAs in their undergraduate years were included. Second, descriptive studies that reported the constructs of medical students regarding EAs were excluded (e.g., perception or motivation about EA participation). Third, articles that did not explicitly explain the learning outcomes of EAs in medical schools were omitted. Screening and reviewing were carried by a team of three researchers, and any disagreement was resolved by discussion. Figure 1 shows the process of screening and selecting articles for review.

## Results

The screening process yielded 64 articles for further review (Table 1). In terms of geographical distribution, we found that approximately 60% of studies (*n* = 38) were conducted in Anglophone countries, including the United States, the United Kingdom, Canada, Dominican Republic, and Australia. The research from Asian countries (*n* = 12), European countries (*n* = 9), South American (*n* = 4), and African country (*n* = 1) followed. Some studies did not specify the academic year of the students, and the number of them. Most of the articles presented a quantitative study (*n* = *46*), where the data were mostly collected via questionnaires. Twelve studies utilized mixed methods, especially qualitative interviews with quantitative questionnaires or qualitative self-reports with quantitative questions. There were six qualitative studies in which the scholars mainly applied the interview method with self-reflection and a qualitative survey. Although no particular chronological trends were detected across the chosen time period, we found that extensive research has been conducted in recent years.

**Table 1:** Description of the articles in the integrative literature review

Author (published year)	Country	Study methods	Participants size	Students’ year
Yusoff et al. (2013) [17]	Malaysia	Quantitative	174	1^st^
Mugerwa et al. (2013) [18]	Uganda	Quantitative	232	N/A
Kost et al. (2014) [19]	USA	Quantitative	663	1^st^ ~ 4^th^
Fang & Lii. (2015) [20]	Taiwan	Quantitative	358	4^th^ ~ 7^th^
Kost et al. (2015) [21]	USA	Quantitative	2,047	N/A
Dekker et al. (2015) [22]	USA	Mixed	31	1^st^, 3^rd^, & 5^th^
Lumley et al. (2015) [3]	UK	Quantitative	4,478	6^th^
Cirone & Saks (2015) [23]	USA	Mixed	204	1^st^ & 2^nd^
Andersen et al. (2015) [24]	Denmark	Quantitative	363	1^st^ ~ 3^rd^
Lattanza et al. (2016) [25]	USA	Quantitative	206	1^st^ & 2^nd^
Greeson, Toohey & Pearce. (2015) [26]	USA	Mixed	44	1^st^ ~ 4^th^
Chapman et al. (2015) [27]	UK	Quantitative	273	All years
Almeida et al. (2015) [28]	Ukraine	Quantitative	140	N/A
Chapman & Bottentuit-Rocha. (2016) [29]	Dominican Rep.	Qualitative	5	N/A
Thoma et al. (2015) [30]	USA	Quantitative	22	1^st^
Fares et al. (2016) [2]	Lebanon	Quantitative	165	1^st^ & 2^nd^
Ayuob, Sayes & Deek. (2016) [31]	Saudi Arabia	Mixed	55	2^nd^ ~ 5^th^
Bridgeman et al. (2016) [32]	UK	Quantitative	50	N/A
Beigy et al. (2016) [33]	Iran	Quantitative	355	3^rd^ & 4^th^
Raulkar & Saks. (2016) [34]	USA	Mixed	223	1^st^ & 2^nd^
Lapinski et al. (2016) [35]	USA	Quantitative	1,294	1^st^ ~ 4^th^
Babenko & Mosewich. (2017) [36]	Canada	Quantitative	640	1^st^ ~ 4^th^
Almalki et al. (2017) [37]	Saudi Arabia	Quantitative	306	1^st^ ~ 4^th^
Bandeali, Chiang & Ramnanan. (2017) [38]	Canada	Mixed	50	1^st^ & 2^nd^
Evans et al. (2017) [39]	USA	Qualitative	14	1^st^ ~ 4^th^
Ranasinghe et al. (2017) [40]	Sri Lanka	Quantitative	471	2^nd^, 4^th^, & 6^th^
Mandich, Erickson & Nardella. (2017) [41]	USA	Qualitative	N/A	N/A
Brandl et al. (2017) [42]	USA	Quantitative	904	1^st^ & 2^nd^
Brown et al. (2018) [43]	Canada	Mixed	123 (survey) & 11 (interview)	1^st^
Whelan, Leddy & Ramnanan. (2018) [44]	Canada	Quantitative	478	1^st^ & 2^nd^
Hsiang et al. (2018) [45]	USA	Qualitative	23	N/A
Bartlett & Huerta. (2018) [46]	USA	Quantitative	22	1^st^ ~ 4^th^
Moglia et al. (2018) [47]	Italy	Quantitative	155	1^st^ ~ 6^th^
Goodcoff et al. (2019) [48]	USA	Quantitative	50	1^st^ & 2^nd^
Bechini et al. (2019) [49]	Italy	Quantitative	100 (pre-lecture survey) & 81 (post-lecture survey)	1^st^, 2^nd^, & 6^th^
Bunting, Saqueton & Batteson TJ. (2019) [50]	USA	Qualitative	11	1^st^
Andreoni et al. (2019) [51]	Brazil	Quantitative	56	N/A
Lodewyk et al. (2020) [52]	Canada	Quantitative	61	3rd
Rigby & Bennett D.D. (2020) [53]	USA	Quantitative	308	All years
Rice et al. (2020) [54]	USA	Mixed	30 (survey) & 5 (interview)	N/A
Valladares-Garrido et al. (2020a) [55]	Latin America	Quantitative	11,587	1^st^ ~ 6^th^
Valladares-Garrido et al. (2020b) [56]	Latin America	Quantitative	11,587	1^st^ ~ 6^th^
Linehan et al. (2020) [57]	Canada	Mixed	136	2^nd^
Bogomolova et al. (2020) [58]	The Netherlands	Quantitative	110	1^st^ & 2^nd^
Ziegler et al. (2020) [59]	Canada	Mixed	25	1^st^
Ayandeh et al. (2020) [60]	USA	Quantitative	44	2^nd^ ~ 4^th^
Shadid et al. (2020) [61]	Saudi Arabia	Quantitative	500	1^st^ ~ 5^th^
Wang et al. (2020) [62]	China	Quantitative	20	N/A
Squeri et al. (2020) [63]	Italy	Quantitative	470	3^rd^ & 4^th^
Ommering et al. (2021) [64]	The Netherlands	Quantitative	315	1^st^
Sepede et al. (2021) [65]	USA	Quantitative	597	1^st^ ~ 4^th^
Izadabadi, Amini & Kiani M. (2021) [66]	Iran	Quantitative	100	1^st^ & 2^nd^
Salih et al. (2021) [67]	Saudi Arabia	Quantitative	40 (control) & 40 (case)	2^nd^ ~ 6^th^
Pang et al. (2021) [68]	USA	Quantitative	688	1^st^ ~ 4^th^
Kuhn et al. (2021) [69]	Germany	Qualitative	20	5^th^ semester
Butcher et al. (2021) [70]	USA	Mixed	864	1^st^ ~ 5^th^
Chang Chan et al. (2021) [71]	Nicaragua	Quantitative	154	3^rd^ & 6^th^
Joshi, Monga & Raina (2022) [72]	India	Quantitative	132	2^nd^
Hayden et al. (2022) [73]	USA	Quantitative	129	2^nd^
Shkola et al. (2022) [74]	Ukraine	Quantitative	60 (control) & 60 (experimental)	1^st^
Diaz (2022) [75]	Australia	Mixed	31	N/A
Domeisen et al. (2022) [76]	USA	Quantitative	56	1^st^ & 2^nd^
Lee et al. (2022) [77]	Canada	Quantitative	313	1^st^ & 2^nd^
Kortz et al. (2022) [78]	USA	Quantitative	59	3^rd^ & 4^th^

**Fig. 2:**
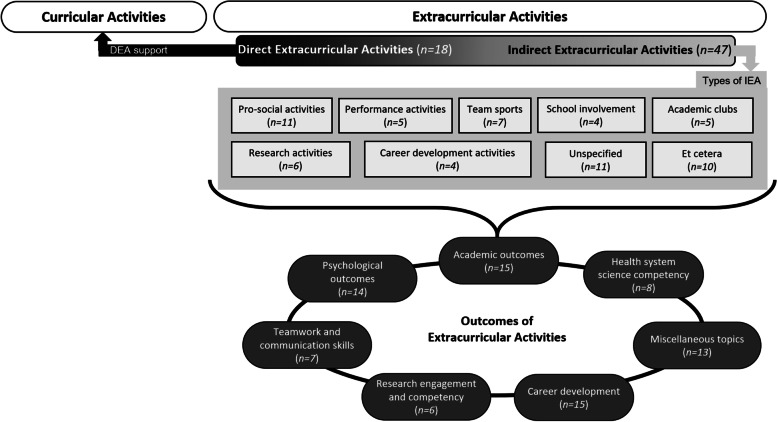
Integrative model of the types and outcomes of extracurricular activities in medical education

For the types of and outcomes of EAs, some articles overlapped in more than one category when they covered several EAs or the EA was multifaceted with various effects (Fig. 2).

### Types of EAs

EAs can be divided into direct and indirect EAs, with the indirect category comprising sub-categories. The research team extracted the types of EAs reported on the target articles and counted the number of targeted articles that belong to each category. A DEA is an EA that is closely related to the learners’ major or formal curriculum, while an IEA is relatively unassociated with them [10]. The former included 18 articles [25, 30, 33, 39, 44, 46, 48, 49, 51, 57–60, 66, 69, 71, 73, 76] and the latter comprised 47 articles.

IEAs contain the characteristics of activities within nine categories (pro-social activities, performance activities, team sports, school involvement, academic clubs, research activities, career development, unspecified, et cetera). The classification of IEAs was based on five criteria from the MSALT [14]. Pro-social activities include social organizations or social events [14] and were discussed in 11 articles [2, 19, 21, 22, 29, 37, 50, 53, 54, 61]. Performance activities indicate artistic activities [14] and comprised five articles [2, 3, 37, 47, 75]. Team sports involve participation in one or more school sports teams [14] and included seven articles [2, 34, 36, 52, 61, 72, 74]. School involvement represents a self-governing body or entertaining club [14] and included four articles [3, 23, 31, 61]. Academic clubs refer to debate or math clubs, or science fairs [14] which do not directly relate to the regular curriculum. These comprised five articles [2, 37, 42, 43, 56]. Activities about research and career development, which are unique traits of medical education, were also added here. Research activities offer students opportunities to be involved directly or indirectly in scientific research like thesis writing, attending academic conferences, and special lectures on research methodology, and included six articles [24, 27, 55, 61, 64, 66]. Career development covers activities to explore career paths as a prospective medical expert and included four articles [21, 23, 25, 32]. Additionally, there were 11 unspecified IEAs that could not be identified in the targeted articles [17, 18, 20, 28, 35, 40, 63, 65, 67, 70, 78]. Ten other non-classified IEAs included counseling [45, 77], teaching [3, 38], mindfulness [26], professional exchanges [62], Asian-American-related EAs [68], video games [47], international exchanges [41], and distinction [23].

### Outcomes of EAs

The research team perused the results and discussion sections of the target articles, extracted the keywords, and coded the keywords into six categories: academic outcomes, health system science (HSS) competency, psychological outcomes, teamwork and communication skills, research engagement and competency, and career development. The keywords that did not belong to any categories above were grouped under “miscellaneous topics.”

#### Academic outcomes

Academic outcomes are further categorized into three domains: knowledge, skill, and attitude. In total, 15 articles reported academic outcomes. Academic knowledge can be measured by test scores or course averages; two studies used a grade point average (GPA) [64, 67] and one used a percentile rank [3]. Most researchers provided a test to measure the students’ knowledge of a specific topic [44, 51, 57, 60, 64, 71, 76] (e.g., anatomic knowledge, ultrasound knowledge). They used pre-and post-tests to evaluate the effect of EAs and found a positive relation between academic performance and EA participation. However, the COMLEX Level 1 performance was not related to the EA [78].

Only three papers reported academic skills as outcomes of EA participation [47, 60, 73], two of which showed that hands-on practice in the extracurricular program was helpful in improving diagnostic reasoning and procedural skills [60, 73]. Academic attitude includes a perceived understanding of the learned knowledge or skills or self-reported confidence about one’s clinical skills [48, 60, 75, 76] and absence at school [72]. Generally, EA participation increased self-reported understanding and confidence. In one study, however, it did not boost confidence in using ultrasound, even though it increased ultrasound knowledge [57].

#### Health system science (HSS) competency

HSS is a framework to understand healthcare as a system focusing on patient care [79]. HSS has six core domains: health care structure and process, health system improvement, value in health care, population, public, and social determinants of health, clinical informatics and health technology, and healthcare policy and economics [79]. Eight articles reported improved competency under health system improvement and population and public health. Under health system improvement, students showcased a more positive attitude toward quality improvement [43, 46] and increased knowledge of healthcare management [45]. Students also learned about underrepresented populations [23, 68], infectious diseases, and preventive strategies [29, 49, 50], which fell under population and public health.

#### Psychological outcomes

14 articles surveyed the psychological states and motivations of students as a potential outcome of EAs. Most of them focused on negative states: stress and burnout [2, 17, 23, 26, 34–36, 39, 61, 65], anxiety, and aggression [17, 20]. Two papers investigated positive states: tolerance of ambiguity and uncertainty [52], and work-life balance [70]. Generally, EA participation alleviated the negative states and increased the positive states. However, the amount of time invested in EAs was not related to burnout and even showed low efficacy [37].

Two papers dealt with motivation, each of which investigated goal orientation and intrinsic motivation. Under goal orientation, engaging in physical activity was positively associated with the mastery approach [36]. Under intrinsic motivation, students reported higher autonomy in extracurricular sessions than in mandatory sessions [30].

#### Teamwork and communication skills

Medical students need to develop teamwork and communication skills as they have to collaborate with other health professionals while attending to patients. Seven articles investigated the effects of EA on teamwork and communication skills. Students who participated in EAs considered teamwork valuable [22], reported increased knowledge, exhibited positive perception of nurses and pharmacists [62], and showed improved cultural competency [41], leadership [31], and communication skills [29, 45, 66].

#### Research engagement and competency

Research engagement is one of the four student engagement domains in health professional education [80]. It is essential for medical students to conduct research and develop research competency considering the importance of evidence-based medicine and the critical role of the physician-scientist [81]. Six articles investigated various variables related to research. One study compared the students’ knowledge of critical thinking and research methods before and after an ultrasound workshop [66]. Two papers measured attitudes toward research and self-perfection about research skills [27, 64], and two studies investigated actual involvement in research, like continuing research in a Ph.D. program and conference attendance [24, 53]. One study reported that EA participation increased both intention and interest in paper publishing and actual paper publishing [55]. EAs were beneficial for increasing research skills and knowledge and cultivating a positive and more enthusiastic attitude toward research.

#### Career development

The careers of most medical students seem to be determined upon their admission to the medical college. However, students require more time to determine their preferred choice of major as they determine their career path. Ten articles investigated the outcomes related to knowledge and attitude about specialized medical fields, and five articles focused on professionalism. Among the articles on career choice, some measured the match rate [19, 21, 25, 77], the students’ interest in and intention to apply to specific majors [25, 32, 54, 59], or the knowledge and perception of the majors [31, 32, 39, 54, 76, 77]. Professionalism was mainly about patient care [22, 54, 75] and medical ethics [33, 69]. These researchers reported that having exposure to specific fields helped students develop a favorable attitude and deepen their understanding of the specialties, which contributed to the development of professionalism.

#### Miscellaneous topics

The outcomes from 13 articles did not fall into the six categories mentioned above. Three articles reported that EA participants could help students manage their time and stress [26, 31, 66]. They found a workshop about stress [66] or leadership [31], and a mindfulness program [26] helped self-management. Five articles investigated the relation between EAs and physical health or physical ability [18, 28, 58, 63, 74], such as the prevalence of tuberculosis infection, vaccination coverage, and visual-spatial abilities. Two articles mentioned time use; participation in academic-scientific programs negatively predicted the use of social networks [56], and students reported that they lost time due to EA participation [23]. Students reported that they could communicate and feel connected with faculty and students by participating in EAs [23, 42]. The last two studies explored what the other researchers had not dealt with; confidence [38] and passion in teaching and emotional intelligence [40], both of which were positively related to EA participation.

## Discussion

This review synthesized the types and effects of EAs in medical colleges. To our knowledge, this is the first attempt to develop an overarching picture of the importance of EAs in undergraduate medical education and their implications. The use of the integrative model in this paper helps in gaining a comprehensive understanding of the role of EAs in colleges with distinctive characteristics of medical education.

We embodied the type of EAs by categorizing unique EAs that were found in medical education. In earlier education studies, EAs were generally classified. Shamsudin et al. (2014) sorted EAs into physical, educational, and social programs [82]. Gilman et al. (2004) differentiated structured collaborative activities from solitary and non-structured activities [83]. Contrarily, the MSALT classification system had the advantage of providing subdivisions based on activity characteristics [84]. However, a concrete EA-type system reflecting the features of undergraduate medical students was needed, in that this classification system was for adolescents. We additionally sorted research activities and career development activities in the process of presenting the integrated model. We expanded the MSALT classification to fit it within the context of medical education.

Our findings about the outcomes of EAs reflect the features of medical education, replicating the prior studies on higher education [85]. Firstly, as reported in previous research, EA participation increased GPA [86, 87]. It also developed medical knowledge, skills, and self-perception of the learned materials and skills. While HSS is considered as a third curriculum [79], concerns have been raised about the feasibility of adding class time for HSS to the regular curriculum due to its hectic nature [88]. We present studies where students can develop their HSS competency in EAs. Secondly, EA studies in medical education focused more on negative states like stress and burnout, while studies on non-medical undergraduate students focused on mental health or well-being [89, 90]. This is likely because stress and burnout are prevalent among medical students [61]. Thirdly, medical students, like engineering students, developed their knowledge, attitude, and skills needed for teamwork [91]. Fourthly, EAs related to scholarly research enhanced students’ research engagement and research competency, which is in line with the article that investigated research engagement in health professional education [80]. In medical education, extracurricular research programs focused on fostering positive research attitudes and increasing research involvement, while mandatory research programs focused on developing research knowledge and skills [92]. Also, medical students deepened their understanding and interest in specific specialties and developed professionalism through EA participation. This may imply that the regular curriculum, including clerkships, provides limited opportunities for exploring specialties. Lastly, the typical outcomes among miscellaneous topics were physical health and physical ability. Only two articles demonstrated a sense of belonging and communication with others as positive outcomes of EA. Medical students’ motivation for EA participation includes networking [1, 93] and interaction with faculty and peers, which has a positive effect on students’ satisfaction and career motivation [94]. These need to be considered as important outcomes in medical education.

Even though we tried our best to comprehensively investigate all apposite studies, the selection criteria might have excluded some relevant articles. That is, we might have missed some relevant studies because of the nature of the search strings used, that is, if the keywords did not appear in the title or abstract. For example, there were no studies found about EA by racial or cultural organizations like Latino Medical Student Association (LMSA), or Student National Medical Association (SNMA), even though they play a huge role in medical education. It might be because research about LMSA or SNMA might not use the keyword “extracurricular activity” as they are big enough not to be named under EA, or it might be a limitation of the database we used as we searched articles on Scopus and PubMed only. Furthermore, this review excluded studies published in languages other than English. Thus, we could not capture the dynamics of EAs in non-English speaking countries; this may lead to an increased risk of bias and limit the generalizability of findings. In the process of deriving an integrative model, we might have missed some distinguishing features of EAs that are specific to the social, institutional, and educational context of individual countries and schools. However, three main researchers took turns examining the articles to derive the most balanced and integrated model.

We would like to propose further research suggestions based on the results and discussion. First, future studies in medical education should prioritize establishing a clear definition of EAs and their subdivisions using standardized categories, based on a thorough analysis of previous studies. This is because the systematization of conceptual definition and classification system guarantees the accumulation of productive knowledge. Second, further research exploring the balanced and effective growth of students’ capacities through EA is needed. We outlined the effects of EAs; EA participation outcomes are related to factors that are rarely covered in the regular curriculum, and the effects of EA can span across several domains. Further research on the usage of EAs for students' holistic development is warranted. Third, follow-up research will be required to overcome the bias toward quantitative research and encourage qualitative research. It is expected that researchers will be able to find the unforeseen aspect and effects of EA through various qualitative methods such as interviews, focus groups, and observational records.

Based on our work, we make the following recommendations for medical educators. First, we recommend that medical educators provide students with diverse and balanced EAs to provide them with rich learning experiences. This is because the outcomes of EAs can be derived differently depending on the EA type. Second, schools need to explain the expected outcomes of EAs and regularly follow up on the students to ensure they benefit from EAs without losing time and stamina. It is difficult to gauge the effects of EAs, as they are not often assessed. Furthermore, it is possible that students feel stressed or lose study time owing to EA participation. Schools can assist the students' choice and participation in EAs by explaining and assessing the effect of EAs.

## Conclusion

This study aimed to collect and synthesize previous articles that dealt with the EAs in undergraduate medical education and their learning impacts, thereby presenting an integrative model of the given topic. Despite a growing emphasis on EAs and the emergence of various types of research, there is no holistic approach to embrace the broadness of previous studies in a collective way. Therefore, this integrative review attempted to expand the understanding of the relevance and implications of EAs to the learning experiences of undergraduate medical students.

EAs in undergraduate medical school were classified into two main categories: DEAs and IEAs. The latter can be further classified into nine categories. It was found that IEA is more widely implemented than DEA, among which pro-social activities ranked at the top, followed by team sports and research activities. Regarding the outcomes of EAs, we identified seven main categories. Most of the selected articles dealt with academic outcomes and career development, which are deeply related to major-specific knowledge, clinical skill, and interest or intention to pursue a certain medical specialty. Psychological outcomes such as burnout and stress represented the second most common category, which reflects the distinctive nature of a medical education context.

## Data Availability

The dataset used during the current study is available from the corresponding author upon reasonable request.

## Abbreviations

EA: Extracurricular Activity
DEA: Direct Extracurricular Activity
IEA: Indirect Extracurricular Activity
MSALT: Michigan Study of Adolescent Life Transitions
HRD: Human Resource Development
GPA: Grade Point Average
HSS: Health System Science
LMSA: Latino Medical Student Association
SNMA: Student National Medical Association
